# Air Travel-Triggered Tension Pneumocephalus Caused by a Frontal Sinus Osteoma: Case Report

**DOI:** 10.3390/reports8010010

**Published:** 2025-01-18

**Authors:** Aleksandar Djurdjevic, Milan Lepic, Jovana Djurdjevic, Svetozar Stankovic, Goran Pavlicevic

**Affiliations:** 1 Clinic for Neurosurgery Military Medical Academy, 11000 Belgrade, Serbiatoza.stankovic@gmail.com (S.S.);; 2 Medical Faculty of the Military Medical Academy, University of Defense, 11000 Belgrade, Serbia; 3 Clinic for Physical Medicine and Rehabilitation, Military Medical Academy, 11000 Belgrade, Serbia

**Keywords:** pneumocephalus, frontal sinus osteoma, spontaneous tension pneumocephalus, air travel, intracranial gas dynamics

## Abstract

**Background and Clinical Significance**: Pneumocephalus, an accumulation of air within the cranial cavity, typically arises from trauma or iatrogenic causes. However, spontaneous occurrences of this are rare and linked to various pathologies affecting the paranasal sinuses, the ear, or the skull base. The impact of air travel on individuals with pneumocephalus remains uncertain despite ongoing research. We report a unique case of spontaneous tension pneumocephalus attributed to a frontal sinus osteoma during air travel. **Case Presentation**: A 55-year-old man presented with headache and dizziness, initiated during a nine-hour international flight two weeks prior. The symptoms abated after landing but recurred on his return flight, accompanied by confusion the following day. A neurological examination revealed no deficits. CT and MRI scans indicated the presence of intraparenchymal air collection in the right frontal lobe, attributed to a frontal sinus osteoma causing a dural tear. Surgical intervention included duroplasty and osteoma removal, with postoperative recovery free of complications. **Conclusions**: Frontal sinus osteoma-induced tension pneumocephalus is exceedingly rare, with only limited cases reported in the literature. This case shows that air travel may exacerbate intracranial gas dynamics that lead to development of tension pneumocephalus with a potentially fatal outcome for patients.

## 1. Introduction and Clinical Significance

Pneumocephalus refers to the accumulation of air within the cranial cavity. Craniofacial trauma is the most common cause of pneumocephalus [1]. Iatrogenic pneumocephalus, often following neurosurgical procedures, is also a frequent occurrence [2]. Spontaneous pneumocephalus is rare and may result from infections, neoplasms, or other pathological processes involving the paranasal sinuses, the ear, or the skull base [1,2,3].

The management of pneumocephalus, regardless of its origin or extent, typically begins with conservative approaches, particularly in cases where the condition is not associated with significant neurological deterioration. Initial treatment focuses on stabilizing the patient’s vital signs and may include bed rest, maintaining a low head position, and administering oxygen therapy to facilitate the reabsorption of air from the intracranial space [4,5]. Studies indicate that approximately 85% of cases resolve spontaneously within 2–3 weeks with conservative management, including analgesics for headache relief and hydration [6,7].

However, surgical intervention may be warranted if the condition progresses to tension pneumocephalus, characterized by a significant mass effect that can lead to neurological deterioration and increased intracranial pressure [8].

The safety of air travel for patients with pneumocephalus remains controversial. Despite many studies published to date, there is no clearly defined opinion on whether it is safe for patients with pneumocephalus to travel by air [9,10,11].

We present an extremely rare case of spontaneous tension pneumocephalus development due to a right frontal sinus osteoma during a flight.

## 2. Case Presentation

A 55-year-old man presented in an emergency department complaining of headache and dizziness. The onset of the symptoms was two weeks earlier during an international nine-hour flight. During the flight, he experienced an intense headache that persisted throughout the flight. After landing, all symptoms resolved. Over the next two weeks, he had no difficulties. On his flight home, the intense headache reoccurred and was accompanied by dizziness. The following day at work, his co-workers observed unusual confusion in his behavior and actions. Concerned about his complaints and behavior, they convinced him to seek medical attention.

The only notable condition in his medical history was hypertension and a surgical correction of a nasal septum deviation performed 10 years earlier. A neurological examination revealed no neurological deficits or mental state alteration.

**Figure 1:**
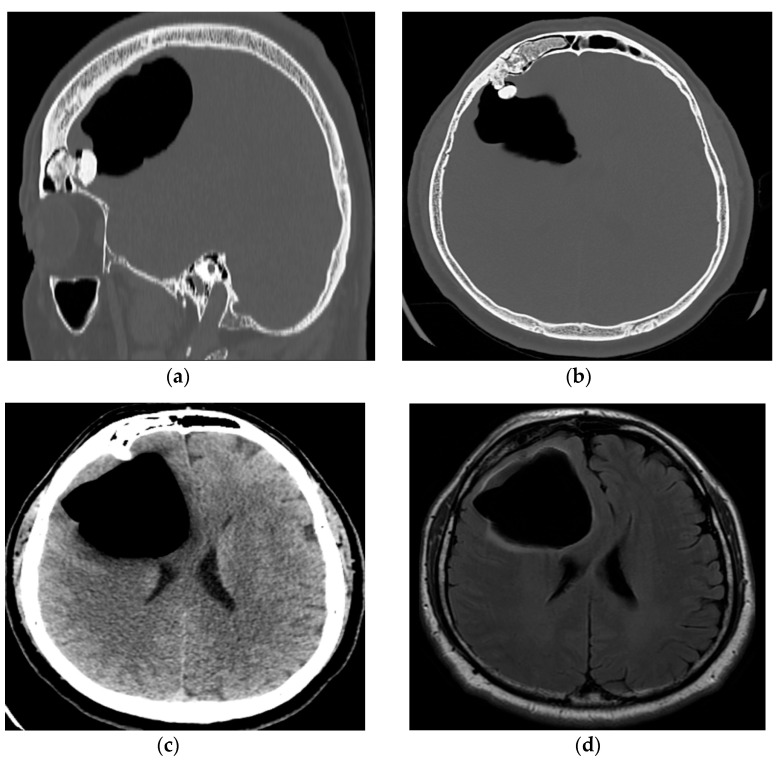
(**a**) A pre-operative sagittal CT scan with a bone window showing the osteoma in the frontal sinus that violates posterior wall of the sinus and protrudes intracranially; (**b**) a pre-operative axial CT scan with a bone window showing the osteoma located in the right frontal sinus protruding intracranially and causing pneumocephalus in right frontal lobe; (**c**) a pre-operative axial CT scan showing the intraparenchymal collection of air in the right frontal lobe that compresses the right lateral ventricle and corpus callosum, causing midsagittal displacement; (**d**) a pre-operative axial T2 Flair MR showing the intraparenchymal collection of air in the right frontal lobe.

CT revealed intraparenchymal air collection in the right frontal lobe that behaved like a mass lesion and exerted compression on the right lateral cerebral ventricle and corpus callosum, causing a midsagittal displacement of 11 mm to the left, as well as the existence of an expansive lesion in the right frontal sinus that compromised the integrity of the frontal bone and protruded intracranially (Figure 1a–c). MRI confirmed the existence of intraparenchymal air collection, with no changes in volume (Figure 1d).

**Figure 2:**
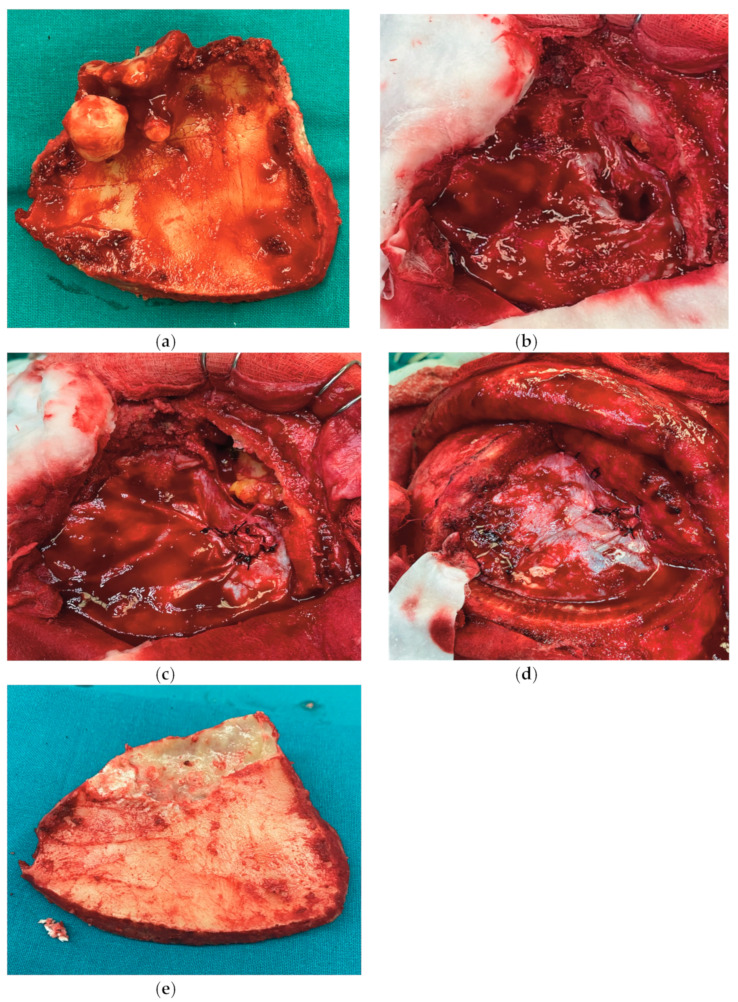
(**a**) Frontal bone with the osteoma attached to the inner side of the frontal sinus; (**b**) dural tear above the frontal cortex caused by osteoma; (**c**) repaired dural defect with autologous muscle graft; (**d**) closure of the frontal sinus using vascularized periosteum; (**e**) frontal bone after osteoma removal.

The operative findings revealed a right frontal sinus osteoma (Figure 2a) that eroded the posterior wall of the frontal sinus and teared the dura (Figure 2b). Duroplasty was performed (Figure 2c), and the frontal sinus was closed using vascularized periosteum (Figure 2d). The osteomas were removed from the frontal bone (Figure 2e) and the bone flap was returned.

**Figure 3:**
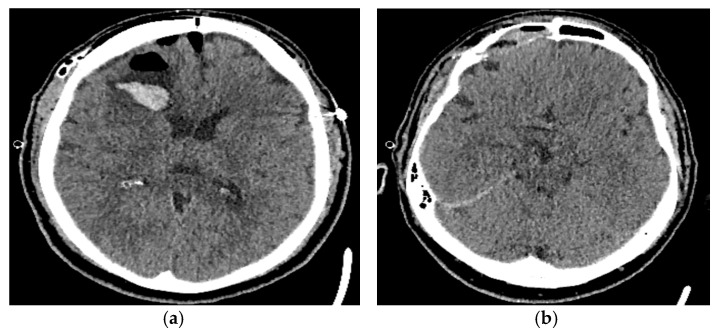
(**a**) A post-operative CT showing the minor collection of air and blood in the operative field; (**b**) a post-operative CT showing the right frontal sinus after the removal of the osteoma.

Postoperatively, the patient did well without any complications. The patient was treated with prophylactic doses of antibiotics during the perioperative period to prevent potential central nervous system infections. Postoperative CT showed a minor collection of air as well as a minor collection of blood in the operative field without midsagittal displacement (Figure 3a,b). The patient was discharged on the seventh postoperative day.

## 3. Discussion

This case underscores the rare occurrence of tension pneumocephalus triggered by a frontal sinus osteoma during air travel. The diagnostic, surgical, and mechanistic insights provided highlight the risks associated with air travel in such patients.

Osteomas of paranasal sinuses are relatively common benign tumors which are usually asymptomatic. Pneumocephalus is a rare complication of osteomas [12]. Two theories explain the formation of pneumocephalus caused by an osteoma. In both cases, it is necessary for the osteoma to break through the bony walls of the cranial cavity and thus create communication between the external environment and the cranial cavity itself. The “ball valve” mechanism was first described by Dandy, who posited that air can enter the cranial cavity through a defect in the dura mater, acting as a one-way valve. This occurs when external pressure exceeds intracranial pressure, allowing air to penetrate into the cranial space. Once inside, the air becomes trapped due to the obstruction created by cranial structures, preventing its escape [13,14,15]. The connection between the intra- and extracranial spaces is essential for the ball valve mechanism to operate effectively. In contrast, the “inverted soda bottle” effect describes a different mechanism where a negative pressure gradient in the cranial cavity, often due to cerebrospinal fluid (CSF) leakage, draws air into the cranial space. This effect occurs when CSF is lost, leading to a vacuum that allows air to replace the lost fluid until the pressures equalize [16,17]. We believe that, in our case, the “ball valve” was the mechanism of pneumocephalus formation. Further, the onset of symptoms in our patient during the flight is likely linked to the impact of air travel on intracranial gas dynamics. Air safety regulations require cabins of commercial aircraft to be pressurized to a standardized minimum of 75 kPa, which is approximately 75% that of atmospheric pressure at sea level (101.3 kPa), at cruising altitude. This creates a hypobaric environment within the aircraft cabin during flight. According to Boyle’s law, the pressure difference between the aircraft cabin and the intracranial air will cause an increase in the intracranial gas volume due to its tendency to equilibrate with the cabin pressure [11]. For individuals with pneumocephalus, this expansion can significantly increase intracranial pressure (ICP), potentially leading to tension pneumocephalus if the air becomes trapped due to a dural defect acting as a one-way valve [18].

We would also like to point out that the patient denied having any fluid leakage from the nose, a salty taste in the throat, or any other symptoms that might suggest cerebrospinal fluid leakage. We believe that the dural tear was small and was compensated by surrounding soft tissue, preventing noticeable CSF leakage under normal atmospheric pressure. However, during flight, the sudden drop in cabin pressure led to rapid intracranial air expansion, triggering clinical symptoms. The absence of observable CSF leakage during the flight could be attributed to the one-way “ball valve” mechanism, which allowed air to enter but not escape, while any minimal CSF loss may have been reabsorbed or not externally apparent.

Spontaneous pneumocephalus associated with air travel is extremely rare. There are only five cases described in the literature to date [19,20,21,22,23]. Among them, in only one case, it is caused by an osteoma [19], but with a different location of the neoplasm and different clinical presentation. We believe this is the first reported case of flight-triggered pneumocephalus caused by a frontal sinus osteoma.

On the other side, there are more studies and case reports that discuss the effect of flying on traumatic or iatrogenically induced pneumocephalus. A significant number of these papers indicate that air travel in a patient with pneumocephalus passes by without any clinical and neurological deterioration [9,23,24,25], which is in contrast to our case. There are also various laboratory experiments that have been performed, which indicate that there is a risk of intracranial gas expansion due to flight, which can lead to increased intracranial pressure [9,11]. Due to such conflicting results and experiences, there is still no clear opinion on whether is it safe for patients with pneumocephalus to fly by plane. Therefore, more work and studies on this topic are needed in the future in order to obtain clearer answers to this question.

Open cranial surgery involving the paranasal sinuses carries a heightened risk of postoperative infections due to the anatomical proximity, potentially leading to severe complications such as meningitis or brain abscesses [26,27]. Prophylactic antibiotics aim to prevent these infections by addressing bacterial colonization in the sinuses. Studies indicate that perioperative antibiotics significantly reduce infection-related complications [27]. However, debates persist regarding the duration and justification of antibiotic use, highlighting the need for further research in this context.

## 4. Conclusions

While pneumocephalus is a recognized complication following trauma or surgical procedures, its spontaneous onset due to a benign sinus tumor, especially in the context of flying, is extremely uncommon. Frontal sinus osteoma-induced pneumocephalus is exceedingly rare, with only limited cases reported in the literature. This case shows that air travel may exacerbate intracranial gas dynamics that leads to the development of tension pneumocephalus, with a potentially fatal outcome for the patients. Given the conflicting data in existing studies regarding the safety of air travel for individuals with pneumocephalus, this case emphasizes the need for a further investigation to establish clear guidelines and safe protocols for managing patients with intracranial air.

## Data Availability

The original contributions presented in this study are included in the article. Further inquiries can be directed to the corresponding author.
